# Potential Value of miR-221/222 as Diagnostic, Prognostic, and Therapeutic Biomarkers for Diseases

**DOI:** 10.3389/fimmu.2017.00056

**Published:** 2017-02-16

**Authors:** Jialin Song, Yuanming Ouyang, Junyi Che, Xiaoming Li, Yi Zhao, Kejia Yang, Xiaotian Zhao, Yinghui Chen, Cunyi Fan, Weien Yuan

**Affiliations:** ^1^Shanghai Jiao Tong University Affiliated Sixth People’s Hospital, Shanghai, China; ^2^Shanghai University of Medicine & Health, Shanghai Sixth People’s Hospital East Campus, Shanghai, China; ^3^School of Pharmacy, Shanghai Jiao Tong University, Shanghai, China; ^4^Department of Neurology, Jinshan Hospital, Fudan University, Shanghai, China

**Keywords:** microRNAs, miR-221/222, diseases, diagnosis, prognosis, therapy, biomarker

## Abstract

microRNAs (miRNAs) are short non-coding RNAs that regulate gene expression by base pairing with their target messenger RNAs. Dysregulation of miRNAs is involved in the pathological initiation and progression of many human diseases. miR-221 and miR-222 (miR-221/222) are two highly homologous miRNAs, and they are significantly overexpressed in several types of human diseases. Silencing miR-221/222 could represent a promising approach for therapeutic studies. In the present review, we will describe the potential value of miR-221/222 as diagnostic, prognostic, and therapeutic biomarkers in various diseases including cancer and inflammatory diseases.

## Introduction

microRNAs (miRNAs), a class of small (19–25 nt in length) single-stranded RNAs, are involved in posttranscriptional regulation of gene expression by base pairing with the complementary sequences in the 3′ untranslated region (3′UTR) of their target messenger RNAs (mRNAs) (1, 2). miRNAs are generated from the transcription of a long precursor (primary miRNA), which is then processed to a stem-loop precursor miRNA (pre-miRNA) by the nuclear protein Drosha in the nucleus (3). The DiGeorge syndrome Critical Region 8 protein (DGCR8) serves as a molecular ruler, directing the cleavage of Drosha (4). The pre-miRNA of 60–100 nt in length is cleaved by endoribonuclease Dicer to mature miRNA and antisense miRNA star products after the transportation of pre-miRNA to the cytoplasm by exportin-5 (5). The mature miRNA is incorporated into a RNA-induced silencing complex, which results in the inhibition of translation or degradation of the target mRNAs by binding to partially complementary sites (6). It has been widely shown that dysregulation of miRNAs is associated with the initiation, development, and progression of cancer or other diseases (7–9). miR-221 and miR-222, encoded in tandem from a gene cluster located on X chromosome (Xp11.3), contain identical seed sequences separated by 727 bases and are highly conserved in vertebrates (10). In healthy conditions, they have been found to regulate essential physiological vascular processes such as angiogenesis, neointimal hyperplasia, vessel wound healing, vascular aging, and atherosclerotic vascular remodeling (11–14). These two highly homologous miRNAs are frequently acting as a gene cluster (miR-221/222) and have been extensively studied in many human malignancies. In this review, we focus on the current knowledge about the roles of miR-221/222 in the diagnosis, prognosis, and therapy for breast cancer, liver cancer, pancreatic cancer, prostate cancer, gastric cancer, colorectal cancer (CRC), glioma, multiple myeloma (MM), and inflammatory diseases (Table 1).

**Table 1:** **Roles of miR-221/222 in human diseases**.

Cancer	Targets	Biological function	Effects following microRNA antisense/inhibitor therapy	Correlation	Reference
Breast cancer	TRPS1, ADIPOR, SOCS1, CDKN1B, ERα, p27, TIMP3	Epithelial-to-mesenchymal transition (EMT), S-phase entry	Restore ERα expression and tamoxifen sensitivity; decrease cell growth; increase apoptosis	Prognosis	(15–20)
Liver cancer	Bone marrow failure, p27, p57, HDAC	Cell growth, apoptosis	Increase apoptotic cell death; decrease tumor nodules	Detection and prognosis	(21–24)
Pancreatic cancer	TIMP-2, PTEN, p27, p57, and p53 upregulated modulator of apoptosis (PUMA), death receptor 5, Bim	Cell proliferation, cell invasion, G1-phase arrest, apoptosis	Decrease cell proliferation	Detection and prognosis	(25–29)
			Antitumor effect		
Prostate cancer	p27, ARHI, SIRT1, HECTD, RAB1A	Cell cycle, apoptosis, androgen receptor signaling, EMT	Decrease tumor growth; increase apoptosis	Tumor stage and prognosis	(10, 30–33)
Gastric cancer	p27, p57, PTEN, RECK	Cell cycle progression	Decrease cell proliferation and migration; increase cell radiosensitivity	Detection and prognosis	(34–38)
Colorectal cancer	p57, RECK, PDLIM2, PTEN	Cell cycle	Decrease proliferation; increase apoptosis; increase radiosensitivity	Detection and prognosis	(39–43)
Glioma	p27, p57, p53, PTPμ, TIMP3	Cell cycle, apoptosis, cell migration, radio-induced DNA damage repair	Decrease tumor growth; increase apoptosis, radiosensitivity	Detection and prognosis	(33, 44–49)
Multiple myeloma	p27, PUMA, PTEN, p57, Ki-67	Cell growth and apoptosis	Antitumor activity, anti-proliferative effect, no organ-related toxicity, restore melphalan sensitivity	Detection and genetic subtype	(50–53)
Malignant melanoma	p27Kip1/CDKN1B and the c-KIT receptor	Differentiation and enhanced proliferation of the melanoma cells	Proliferation, differentiation, apoptosis, and angiogenesis	Detection and prognosis	(54, 55)
	PI3K/AKT				
Rheumatoid arthritis	MMP-3, MMP-9, vascular endothelial growth factor	Cell growth	Inhibit pro-inflammatory cytokines and cell invasion; increase cell apoptosis	Detection	(56, 57)
Atherosclerosis	PGC-1a, AdipoR1	Reactive oxygen species (ROS), cell apoptosis, NO synthesis	Ameliorate inflammation-induced cellular ROS production	Detection	(58–61)

## Breast Cancer

In basal-like breast cancer, stimulated transcription of miR-221/222 by basal-like transcription factor FOSL1 (Fos-like 1, also named Fra1) can promote epithelial-to-mesenchymal transition (EMT) *via* targeting the 3′UTR of trichorhinophalangeal 1 (TRPS1), leading to increased cell migration and invasion (15). Moreover, overexpressed miR-221/222 can promote the EMT in breast cancer by negatively regulating adiponectin receptor 1 (ADIPOR1) (16). Li et al. also reported that a significantly higher expression of miR-221/222 was observed in highly invasive basal-like breast cancer, which could contribute to S-phase entry, cellular migration, and invasion through the downregulation of suppressor of cytokine signaling 1 (SOCS1) and cyclin-dependent kinase inhibitor 1B (CDKN1B) (17). Studies also showed that miR-221/222 enhanced breast cancer growth, migration, and invasion, meanwhile propagating the self-renewal of breast cancer stem cells by targeting phosphatase and tensin homolog/Akt (PTEN/Akt) pathway (62).

Overexpression of miR-221/222 can contribute to estrogen-independent growth and fulvestrant resistance in breast cancer, which occurs together with β-catenin activation, dysregulation of transforming growth factor beta (TGF-β)-induced growth inhibition and p27 suppression (63). Increased tamoxifen resistance in breast cancer is associated with the inhibition of p27 regulated by elevating the expression of miR-221/222, and overexpression of p27 in the resistant cells can enhance their sensitivity to tamoxifen (64). Moreover, it has been reported that miR-221/222 negatively regulates estrogen receptor alpha (ERα), and knockdown of miR-221/222 can partially restore ERα expression and tamoxifen sensitivity (65). Downregulated expressions of p27 and ERα can enhance tamoxifen resistance by secreting miR-221/222 in exosomes for ER-positive and tamoxifen-sensitive breast cancer cells (66). Inhibition of miRNA-221/222 in ER-positive human breast adenocarcinoma cell line (MCF-7) can also increase the sensitivity to tamoxifen through the upregulation of tissue inhibitor of metalloproteinases-3 (TIMP3) (18). Therefore, miR-221/222 might serve as potential therapeutic targets for drug resistance in breast cancer.

Nucleolin, an integral component of the microprocessor complex consisting of Drosha and DGCR8, can regulate the expression of several miRNAs (such as miR-21, miR-221/222, and miR-103) that involved in breast cancer aggressiveness and drug resistance (63, 67–70). A specific aptamer AS1411 has been demonstrated to be able to modulate nucleolin binding to the microprocessor complex and affect the expressions of these above miRNAs by posttranscriptional regulation, thus reducing breast cancer metastasis both *in vitro* and *in vivo* (19). Moreover, nucleolin targeting treatment could also lead to decreasing cell growth and increasing apoptosis of fulvestrant-resistant breast cancer cells, thereby suggesting that targeting compounds of nucleolin has the potential to improve the sensitivity of drug resistant breast cancer cells in the clinical practice (19). Falkenberg et al. have reported that high expression of miR-221/222 is significantly associated with the occurrence of distant metastases in breast cancer, thereby suggesting that miR-221/222 may become promising markers for breast cancer prognosis (20).

## Liver Cancer

Upregulated expression of miR-221/222 is involved in the progression of liver fibrosis by the activation of stellate cells (71). Moreover, miR-221/222 overexpression is linked to liver tumorigenesis from normal liver through cirrhosis to full-blown hepatocellular carcinoma (HCC), and miR-221/222 can enhance cell growth by targeting the cyclin-dependent kinase inhibitor p27 (72, 73).

Many studies have demonstrated that miR-221 can increase cell growth, inhibit apoptosis, and promote tumor progression in HCC (72, 74, 75). miR-221 overexpression is able to inhibit apoptosis of HCC-derived cell lines by targeting bone marrow failure (BMF) syndromes, and increased apoptotic cell death can be caused by miR-221 silencing (21). The hypothesis that increased expression of miR-221 might posttranscriptionally downregulate BMF to affect the TGF-β proapoptotic signals will require further investigations (76, 77). The increased expression of miR-221 contributes to the progression of liver cancer by downregulating the histone deacetylase (HDAC) through natural killer/cells-Jun (NK/c-Jun) activation and nuclear factor-kappa B p65 (NF-κBp65) nuclear translocation (22). Increase in miR-221 expression and repression of its target genes (such as proapoptotic BMF and cyclin-dependent kinase inhibitor p27/57) have been revealed in the liver of a miR-221 transgenic mouse model, and *in vivo* delivery of anti-miR-221 oligonucleotides can lead to significant reduction of the number and size of tumor nodules (23). The DNA hypomethylation at miR-221/222 locus and wild-type tumor repressor protein 53 (TP53) activation by targeting MDM2 (E3 ubiquitin protein ligase homolog) is confirmed to be contributors to miR-221 overexpression in clinical specimens of HCC (78). It has been found that miR-221 is a core miRNA, which targets lots of HCC-related genes by feed-forward regulatory loops combining transcription factors from bioinformatics analysis, and lentivirus-mediated miR-221 silencing can significantly suppress liver cancer cells as well as the growth of hepatoma xenografts *in vivo* (79).

Overexpressed miR-221 in serum samples of HCC patients correlates with tumor size, cirrhosis, tumor stage, and overall survival rate (24). An obvious relationship has been revealed between miR-221 levels in tissues and the migration, invasion, metastasis, tumor capsular infiltration, and time to recurrence after surgical resection of HCC (21, 74). Hence, miR-221 may be valuable for the prognosis of HCC patients and the use of miRNA silencing technology appears to be a promising therapeutic approach for HCC.

## Pancreatic Cancer

Overexpressed miR-221/222 can contribute to the expression of matrix metalloproteinases-2 (MMP-2) and MMP-9 by directly targeting tissue inhibitor of MMP-2 (TIMP-2), thereby facilitating pancreatic cancer invasion (25). Transfection of pancreatic cancer cells with miR-221 inhibitor could inhibit cell proliferation by upregulation of phosphatase and tensin homolog (PTEN), p27, p57, and p53 upregulated modulator of apoptosis (PUMA) (26). Combination of chemotherapeutic drug sunitinib at low dosage and anti-miRNA oligonucleotides targeting the overexpressed miR-21, miR-221/222, and miR-10 can achieve notable synergistic antitumor effect in pancreatic ductal adenocarcinoma cells, thereby indicating that this combinatory approach might be of great importance for therapeutic applications in this disease (27). Moreover, Tanaka et al. reported that metformin could suppress the expression of miR-221, which was responsible for G1-phase arrest and apoptosis *via* the upregulation of p27, death receptor 5, and Bim in pancreatic cancer cells (28).

In the specimens of pancreatic cancer tissue, miR-221 and miR-222 were confirmed to be significantly upregulated, thereby suggesting that these two miRNAs had the potential to be used as diagnostic biomarkers for pancreatic cancer (80, 81). In addition, elevated expression of miR-222 has been reported to be an independent predictor of poor prognosis of pancreatic cancer (29).

## Prostate Cancer

It was reported that overexpression of miR-221/222 might contribute to the progression of prostate carcinoma through downregulating cell cycle inhibitor p27 (10) and aplasia ras homolog member I (ARHI), which influenced cell cycle and apoptosis (82, 83). Yang et al. observed an increase in SIRT1, cell proliferation, and apoptosis and decrease in migration reduction after transfecting PC-3 cells with miR-221/222 inhibitor, thereby suggesting that the tumor-promoting role of miR-221/222 might be mediated by the activation of silent information regulator two homolog one (SIRT1) (30). The *in vivo* study by Mercatelli et al. also confirmed that miR-221/222 inhibition could lead to the upregulation of p27 and reduce the tumor growth of pre-established prostate carcinoma xenografts, thereby indicating the therapeutic potential of miR-221/222 in prostate cancer (31). Wang et al. found that miR-221/222 promoted cell proliferation and repressed apoptosis of prostate cancer cells by repressing caspase-10, and prostate cancer cells were sensitized to tumor necrosis factor-a/cycloheximide (TNF-α/CHX)-induced apoptosis after miR-221/222 knockdown (84).

Androgen receptor (AR) signaling has a critical role in the development and progression of prostate cancer (85). Many studies have found that miR-221/222 is important in tumorigenesis and progression from androgen-dependent to androgen-independent (castration-resistant) prostate cancer (86, 87). Upregulation of miR-221/222 has been observed in the tumor tissues of patients with castration-resistant prostate cancer (88). Moreover, miR-221/222 was involved in the progression from hormone-sensitive to castration-resistant prostate cancer by downregulating HECTD2 and RAB1A, which subsequently led to reprograming of AR signaling, and activation of EMT and new cyclins (32).

The levels of miR-221/222 were significantly higher in aggressive prostate cancer tissue samples than in non-aggressive prostate cancer tissue samples (83). It has been concluded that elevated miR-221/222 expression is significantly associated with a poor overall survival for patients with prostate cancer (33).

## Gastric Cancer

The expression of miR-222/221 is significantly higher in gastric tumor samples compared to the corresponding normal tissues, and miR-222/221 can enhance gastric tumor growth in the mouse xenograft model (34). Meanwhile, miR-221/222 can facilitate the cell cycle progression of gastric cancer cells by downregulating p27 and p57 (34). In addition, it has been indicated that increased miR-221/222 can significantly inhibit reversion-inducing cysteine-rich protein with kazal motifs (RECK) whose aberrant methylation is useful for early diagnosis and treatment of peritoneal metastasis of gastric cancer, thereby resulting in enhanced proliferation and invasion of gastric cancer cells (35, 89). Knockdown of miR-221/222 with anti-miRNA oligonucleotides can inhibit cell growth, suppress cell invasion, and enhance cell radiosensitivity of human gastric cancer cells through the upregulation of PTEN (36). Xu et al. reported that antisense oligonucleotide targeting miR-21, miR-106a, and miR-221 could effectively inhibit the proliferation and migration of human gastric cancer cells (90). Therefore, modulation of miR-221/222 expression by antisense nucleic acid technology might be helpful for gene therapy of gastric cancer.

The increased expression of miR-221 in gastric cancer tissue samples is associated with advanced clinical stage, local invasion, lymphatic metastasis, and poor survival (91). The serum miR-221 has been identified to be a potential biomarker for early detection of gastric cancer and significantly positive correlated with poor differentiation of gastric cancer according to a population-based study (37). Furthermore, miR-222 in plasma is significantly correlated with clinical stages, lymph nodes metastasis, and overall survival (38). These data indicate that circulating miR-221/222 has potential diagnostic and prognostic values in gastric cancer.

## Colorectal Cancer

Sun et al. have demonstrated that miR-221 significantly overexpressed in 90% of CRC samples, and the miR-221 expression is positively correlated with an advanced tumor node metastasis stage and local invasion (39). They also confirmed that miR-221-specific inhibitor could markedly inhibit CRC cell proliferation and induce apoptosis by upregulating cyclin-dependent kinase inhibitor/p57 (CDKN1C/p57) (39). Moreover, miR-221 overexpression enhanced CRC cell invasion and metastasis through targeting RECK (40). It has been reported that miR-221/222 can reduce the ubiquitination and degradation of RelA and activator of transcription 3 (STAT3) proteins by directly binding to 3′UTR of PDLIM2, and miR-221/222 inhibitors can reduce CRC cell proliferation and colony formation (41). Formation of fewer tumors was observed after the injection of lentiviruses expressing miR-221/222 sponges (miR-221/222) in the mice model of colitis. As a competitive inhibitor of small RNAs in mammalian cells, miRNA sponges stably expressed miR-221/222 leading to reduced tumor volume and weight, as well as fewer Ki67-positive cells in tumors (41, 92). Moreover, inhibition of miR-221 could enhance the radiosensitivity of CRC cells by upregulating PTEN (42).

According to the study by Pu et al., plasma miR-221 is a potential diagnostic biomarker for differentiating CRC patients from controls and elevated plasma miR-221 level is a significant prognostic factor for poor overall survival in CRC patients (43). A survival analysis has also indicated that high expression of miR-221 in colon cancer is closely associated with a shorter survival time (93). The levels of miR-221 in stool samples from subjects with CRC were significantly higher, and the area under the curve value of stool miR-221 was 0.73, thereby suggesting that stool-based miR-221 might be used as a potential detection biomarker for CRC (94).

## Glioma

The enhanced expression of miR-221/222 in glioblastoma can promote S-phase entry by targeting the cell cycle inhibitors (p27 and p57), and knockdown of miR-221/222 strongly reduced tumor growth *in vivo* (44, 45). Upregulation of miR-221/222 inhibits cell apoptosis by targeting PUMA in human glioma cells (46). Moreover, miR-222/221 overexpression can promote cell migration and growth by downregulating the protein tyrosine phosphatase μ (PTPμ) in glioblastoma (47, 95, 96). It was also found that miR-221/222 could regulate the invasion capability of glioma cells by directly targeting TIMP3 (33). Zhang et al. also reported that the plasma miR-221/222 family levels were found to be significantly upregulated in glioma patients, and high positive plasma miR-221 and miR-222 were both correlated with poor survival rate, which should be considered as a new additional tool to better characterize glioma (97).

Repression of miR-221 by a peptide nucleic acid could contribute to growth inhibitory effects *via* significant increase in p27 and TIMP3, thereby suggesting that sequence-selective targeting against miR-221 might have the potential for the treatment of human gliomas (98). Recently, administration of temozolomide in combination with radiotherapy was the best clinical approach for glioblastoma treatment (99, 100). Knockdown of miR-221/222 sensitized glioma cells to temozolomide and increased apoptosis in human gliomas by regulating apoptosis independent of the p53 status (48). The response to temozolomide is dependent on the intracellular level of the alkylating enzyme O^6^-methylguanine–DNA methyltransferase (MGMT), and Quintavalle et al. have showed that downregulation of MGMT targeted by miR-221/222 may render cells unable to repair their genetic damage (101). It suggested that miR-221/222 could serve as potential therapeutic targets for radioresistance of glioblastoma cells since miR-221/222 played a crucial role in radio-induced DNA damage repair and silencing of miR-221/222 could significantly increase radiosensitivity of glioblastoma cells by PTEN-independent inhibition of Akt (49). Moreover, the combined anti-miR-221/222 and radiotherapy is more efficient for the suppression of tumor growth than anti-miR-221/222 or radiotherapy alone in a murine glioblastoma model (49).

The expression of miR-221/222 in high-grade gliomas is significantly increased compared with low-grade gliomas, and increased level of miR-221/222 is positively correlated with the degree of glioma infiltration that is associated with poorer overall survival (33). The study by Zhang et al. have demonstrated that plasma miR-221/222 levels are significantly upregulated in glioma patients and high plasma levels of miR-221/222 correlate with poor survival rate, thereby suggesting that miR-221/222 should be considered as prognostic biomarkers for glioma (97).

## Multiple Myeloma

The expression of miR-221/222 was revealed to be upregulated in human myeloma cell lines and primary MM tumors, and miR-221/222 of plasma cells was at higher levels in t(4;14) patients than in the other classes (102, 103). Silencing miR-221/222 by specific inhibitors could result in a powerful antitumor activity in MM cells bearing t(4;14) and murine models of human MM *via* upregulation of p27, PUMA, PTEN, and p57 (50). At the same time, the antitumor activity of miR-221 inhibitors was higher than that of miR-222 inhibitors (50). The 13-mer locked nucleic acid-inhibitor-miR-221 (LNA-i-miR-221) had remarkable anti-proliferative effect on t(4;14)-translocated MM cells *via* strong derepression of p27, and significant antitumor activity against t(4;14) MM xenografts was also observed after 2 weeks of exposure to LNA-i-miR-221 with upregulated p27 and reduced Ki-67 (51). Meanwhile, neither behavioral changes nor organ-related toxicity was observed in mice after the exposure to LNA-i-miR-221 according to the pathology examination (51).

Moreover, it is urgently needed to develop novel therapeutic strategies for MM since this disease can commonly progress to drug-refractory end stage that remains an obstacle to long-term survival (104, 105). The expression level of miR-221/222 is negatively correlated with the melphalan sensitivity of MM cells, and miR-221/222 inhibition restores melphalan sensitivity and triggers apoptosis of MM cells with upregulation of proapoptotic Bcl-2-binding component 3 (BBC3)/PUMA, modulation of drug influx–efflux transporters SLC7A5/LAT1, and the ATP-binding cassette transporter multidrug resistance-associated protein 1 (ABCC1/MRP1) (52). The combined treatment of severe combined immunodeficient/non-obese diabetic mice bearing human melphalan-refractory MM xenografts with LNA-i-miR-221 and melphalan can also overcome drug resistance and induce antitumor activity, which is associated with upregulation of BBC3/PUMA and downregulation of ABCC1 (52). Therefore, the miRNA-based therapies may be useful for clinical trials to overcome resistance in drug-refractory MM, especially miR-221. The miR-221 is confirmed to be significantly upregulated in the plasma samples of MM patients, and miR-221 is associated with chromosomal abnormalities, thereby suggesting that it has potential to be considered as clinical biomarkers for MM (53).

## Malignant Melanoma (MML)

Malignant melanoma is a fatal and aggressive neoplasm. miR-221 is an abnormally expressed in MML cells, and it induces the malignant phenotype through downmodulation of p27Kip1/CDKN1B and the c-KIT receptor; and the significantly increased level of miR-221 in patients’ circulating serum with MML is used as a new tumor marker, which is useful for the MML diagnosis, the *in situ* differentiating MML from stages I to IV MML, evaluating cancer progression, and monitoring patients during the follow-up period. In addition, the levels of miR-221 in serum are correlated with cancer thickness; miR-221 will be useful as a prognostic marker for MML patients (54). miR-222 in exosome can mediate transfer and sufficiently increase MML malignancy (55).

## Inflammatory Diseases

The miR-221/222 is also involved in the pathogenesis of some inflammatory diseases, such as rheumatoid arthritis and atherosclerosis. The miR-221/222 has been found to be overexpressed in rheumatoid arthritis synovial fibroblasts, serum, and synovial tissues of patients with rheumatoid arthritis (56, 57). Furthermore, downregulation of miR-221 in fibroblast-like synoviocytes could significantly inhibit the expression of pro-inflammatory cytokines and chemokine, inhibit cell migration and invasion, and induce cell apoptosis *via* inhibiting vascular endothelial growth factor (VEGF), matrix metalloproteinase-3 (MMP-3), and matrix metalloproteinase-9 (MMP-9) (56). The overexpression of miR-221 can also decrease the level of BMP2, p-Smads, and osteogenic genes in degenerated annulus fibrosus cells (106). Overexpression of miR-221-inhibited adiponectin-stimulated nitric oxide (NO) in HUVECs indicated that miR-221 targeted AdipoR1 to regulate endothelial inflammatory response (58).

Serum miR-221/222 level significantly changed in patients with carotid atherosclerosis and coronary artery disease, thereby suggesting that they might be potential diagnostic biomarkers (59, 60). Xue et al. have found that overexpression of miR-19b and miR-221/222 in human aortic endothelial cells can induce reactive oxygen species (ROS) overproduction, which eventually leads to cell apoptosis and contributes to the pathogenesis of atherosclerosis (61). Moreover, inhibition of miR-19b and miR-221/222 expression with miRNA inhibitors can ameliorate the inflammation-induced cellular ROS production by regulating peroxisome proliferator-activated receptor-γ coactivator-1α (PGC-1α), thereby suggesting that these three miRNAs may be the potential therapeutic targets for atherosclerosis and coronary restenosis (61). The miR-221/-222 cluster orchestrates the antiviral and inflammatory immune response to viral infection of the heart, which demonstrated that the inhibition of miR-221/-222 increases viral load, inflammation, and overall cardiac injury upon viral myocarditis (107). It has been reported that miR-221 can suppress NO synthesis and activate nuclear factor-kappa B (NF-κB) signaling in human umbilical vein endothelial cells by targeting adiponectin receptor (AdipoR1), thereby suggesting that miR-221 might be involved in regulating endothelial inflammation response in vascular diseases (such as atherosclerosis) (58).

## Discussion

The discovery of the important roles of miR-221/222 in cancer and inflammatory diseases has shown great potential in both basic research and clinical applications. As one of the cancer biomarkers, miR-221/222 should be given more attention because of its wide existence and high correlation with cancer and inflammatory diseases. In this review, we have focused on the recent advances related to miR-221/222 for the diagnosis, prognosis, and therapy in breast cancer, liver cancer, pancreatic cancer, prostate cancer, gastric cancer, CRC, glioma, MM, MML, and inflammatory diseases.

**Figure 1:**
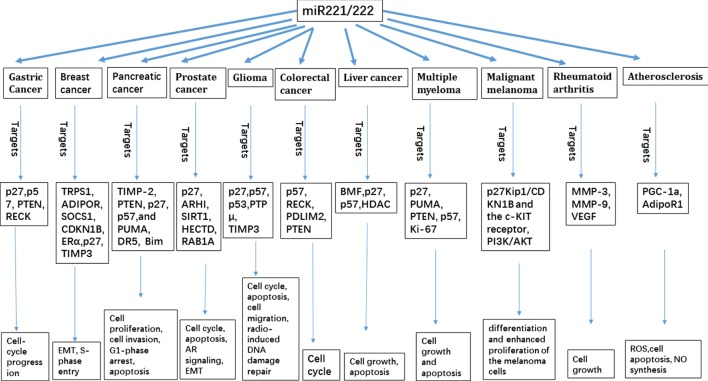
**Schema summarizing the different cellular pathways targeted by miR-121/122**.

miR-221/222 is overexpressed in breast cancer, liver cancer, pancreatic cancer, prostate cancer, gastric cancer, CRC, glioma, MM, and MML (54). Furthermore, miR-221/222 is almost undetectable in normal human melanocytes and is increasingly expressed in the cancers (54). miR-221/222 may mediate the functions of cancer cells to proliferate, differentiate, and invade (Figure 1). Therefore, high levels of miR-221/222 in serum may be useful as a prognostic marker and develop some new diagnosis and prognosis methods for the cancers; and inhibition or silence miR-221/222 may develop some treatment methods for the cancers.

Inflammation is causally linked to numerous clinical pathologies, including cancer, heart disease, and type 2 diabetes. Pro-inflammatory macrophages have been widely implicated in the initiation and progression of these diseases and therefore are a key target for therapeutic intervention. miR-221/222 families play crucial roles in these diseases through different signaling pathways by regulating the expression of different proteins. For example, miR-221/222 regulates endothelial nitric oxide synthase protein levels after Dicer silencing targeting c-Kit for the inhibition of angiogenesis. Autophagic signaling networks are modulated by miR-221/222 in cancer. In inflammatory diseases, miR-221/222 expression leads to the inhibition of VEGF, MMP-3, and MMP-9; ROS overproduction by regulating PGC-1α; suppression of NO synthesis; and activation of NF-κB signaling by targeting AdipoR, thereby regulating the endothelial inflammatory response. Since miR-221/222 families have extensively been studied in cancer network and inflammatory diseases, we consider that miR-221/222 acts as promising biomarkers for cancer and inflammatory diseases and it would offer a new way in molecular targeting cancer treatment. Like other miRNAs, the miR-221/222 cluster plays a remarkable role in cancer and inflammatory diseases. Due to the complexity in diagnosis and treatment of these diseases, miR-221/222 has great potential value as a new biomarker. A combined use of miR-221/222 and other tumor biomarkers will maximum benefit the diagnosis, prognosis, and treatment.

The serum level of miR-221/222 will be useful as a prognostic marker and develop some new diagnosis and prognosis methods for some cancers and inflammatory diseases; if the serum level of miR-221/222 significantly increases than in healthy controls, they should be considered cancer candidates; and inhibition, control, or silence miR-221/222 will develop some treatment methods for the cancers.

## Conclusion

In this review, we focused on each disease for a comprehensive analysis, which is different from previous reviews. Current results in the literature suggest that miR-221/222 has the potential to be considered as novel diagnostic and prognostic biomarkers for these diseases. Moreover, particular attention should be given to the gene therapies based on miR-221/222 for the development of novel therapeutics for cancer and inflammatory diseases.

## Author Contributions

WY, JS, CF, and YC conceived and participated in its design, searched databases, extracted and assessed studies, and helped to draft the manuscript. JS, YO, JC, XL, YZ, KY, XZ, YC, WY, and CF participated in the conceptualization, data extraction, and analysis. JS wrote the manuscript, and WY conceived the initial idea and the conceptualization, participated in the data extraction and analysis, and revised the manuscript. All the authors read and approved the final manuscript.

## Conflict of Interest Statement

The authors declare that the research was conducted in the absence of any commercial or financial relationships that could be construed as a potential conflict of interest.
